# Gaseous Mediators and Mitochondrial Function: The Future of Pharmacologically Induced Suspended Animation?

**DOI:** 10.3389/fphys.2017.00691

**Published:** 2017-09-19

**Authors:** Clair Hartmann, Benedikt Nussbaum, Enrico Calzia, Peter Radermacher, Martin Wepler

**Affiliations:** ^1^Institute of Anesthesiological Pathophysiology and Process Engineering, Ulm University Hospital Ulm, Germany; ^2^Department of Anesthesiology, Ulm University Hospital Ulm, Germany

**Keywords:** gasotransmitters, mitochondria, reactive oxygen species, suspended animation, hibernation

## Abstract

The role of nitric oxide (NO), carbon monoxide (CO), and hydrogen sulfide (H_2_S) as poisonous gases is well-established. However, they are not only endogenously produced but also, at low concentrations, exert beneficial effects, such as anti-inflammation, and cytoprotection. This knowledge initiated the ongoing debate, as to whether these molecules, also referred to as “gaseous mediators” or “gasotransmitters,” could serve as novel therapeutic agents. In this context, it is noteworthy, that all gasotransmitters specifically target the mitochondria, and that this interaction may modulate mitochondrial bioenergetics, thereby subsequently affecting metabolic function. This feature is of crucial interest for the possible induction of “suspended animation.” Suspended animation, similar to mammalian hibernation (and/or estivation), refers to an externally induced hypometabolic state, with the intention to preserve organ function in order to survive otherwise life-threatening conditions. This hypometabolic state is usually linked to therapeutic hypothermia, which, however, comes along with adverse effects (e.g., coagulopathy, impaired host defense). Therefore, inducing an on-demand hypometabolic state by directly lowering the energy metabolism would be an attractive alternative. Theoretically, gasotransmitters should reversibly interact and inhibit the mitochondrial respiratory chain during pharmacologically induced suspended animation. However, it has to be kept in mind that this effect also bears the risk of cytotoxicity resulting from the blockade of the mitochondrial respiratory chain. Therefore, this review summarizes the current knowledge of the impact of gasotransmitters on modulating mitochondrial function. Further, we will discuss their role as potential candidates in inducing a suspended animation.

## Introduction

**Figure 1:**
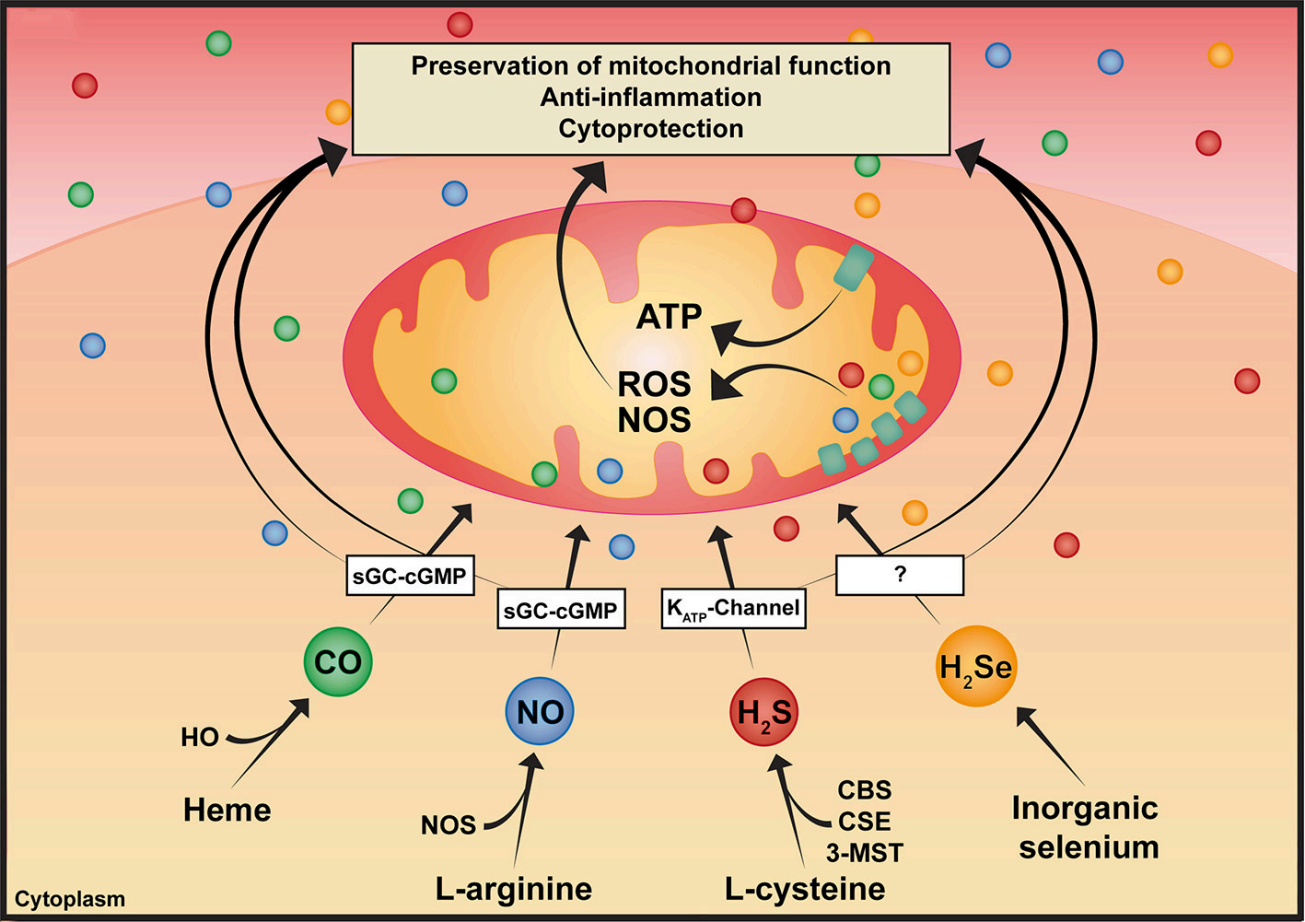
Pleiotropic effects of gaseous mediators. Simplified illustration of the synthesis and various effects of gaseous mediators: CO is enzymatically produced as a result of heme degradation via the family of HO. NO is produced by three NOS isoforms. H_2_S is synthesized by three enzymes, CBS and CSE, which are predominantly cytosolic, and 3-MST, which is responsible for the majority of mitochondrial H_2_S production. As illustrated in the scheme, gaseous mediators share some common features, such as modulating mitochondrial bioenergetics, anti-inflammatory and cytoprotective effects, among others. CBS, cystathionine-β-synthase; CO, carbon monoxide; CSE, cystathionine-γ-lyase; H_2_S, hydrogen sulfide; H_2_Se, hydrogen selenide; HO, heme oxygenase; 3-MST, 3-mercaptopyruvate-sulfurtransferase; NO, nitric oxide; NOS, nitric oxide synthase.

Nitric oxide (NO, Table 1), carbon monoxide (CO), and hydrogen sulfide (H_2_S), are gases referred to as “gaseous mediators” or “gasotransmitters” (Szabó, 2010). Albeit all being toxic at high concentrations, they are biologically active molecules, in particular, at low concentrations. Gasotransmitters share significant similarities: they are produced endogenously, they easily diffuse through cell membranes due to their low molecular weight and thus, are independent of membrane-bound receptors (Figure 1). Consequently, they are ubiquitous signaling mediators with widespread (patho-)physiological properties. Due to their anti-inflammatory and cytoprotective functions, they have been applied as therapeutic targets in various shock models. Most importantly in this context, NO, CO, H_2_S [and possibly also hydrogen selenide (H_2_Se)], can modulate mitochondrial function, despite targeting different sites within the mitochondrion. In line with this, multiple studies have indicated their potential to induce “suspended animation,” a metabolic state characterized by the downregulation of metabolic pathways.

**Table 1:** List of abbreviations and definitions.

**Abbreviations**	**Definitions**
ALI	Acute lung injury
ARDS	Acute respiratory distress syndrome
ATP	Adenosine triphosphate
CO	Carbon monoxide
CO_2_	Carbon dioxide
COHb	Carboxyhemoglobin
CORMs	CO-releasing donors
COX	Cytochrome c oxidase
ETC	Electron transport chain
FADH_2_	Flavin adenine dinucleotide
H_2_S	Hydrogen sulfide
H_2_Se	Hydrogen selenide
I/R	Ischemia/reperfusion
MAPK	Mitogen-activated protein kinase
NADH	Nicotamide adenine dinucleotide
NaSeH	Sodium hydrogen selenide
NO	Nitric oxide
O_2_	Oxygen
ONOO^−^	Peroxynitrite
ppm	Parts per million
RNS	Reactive nitrogen species
ROS	Reactive oxygen species
SQR	Sulfide quinone reductase

*Table presents a summary of the abbreviations used throughout the manuscript including the respective definitions*.

Suspended animation resembles hibernation (and/or estivation) and is characterized by “the slowing down of life processes by external means without termination” (Asfar et al., 2014) in order to survive otherwise life-threatening conditions, e.g., extreme temperatures, prolonged reduction of O_2_ supply, and is associated with decreased energy expenditure and a reduced body temperature. From a clinical perspective, the controlled induction of a hypometabolic state in order to hibernate the whole organism and/or isolated organs could improve outcome by prolonging tolerance against ischemia/reperfusion (I/R) injury (Roth and Nystul, 2005). This concept of “Buying time in suspended animation” emerged more than a century ago, and since, several studies have described the successful induction of a suspended animation-like state, e.g., by O_2_ deprivation (Hochachka et al., 1996), hyperbaric hyperoxia (Richards et al., 1963), and magnesium (Webb et al., 1966). However, inducing suspended animation is usually linked to target temperature management (e.g., therapeutic hypothermia). Clearly, organ-protective effects have been reported not only in primitive life forms, but also in mammals (Richards et al., 1963). Therefore, target temperature management is part of the guidelines of the management after cardiac arrest; this however, may involve severe adverse effects, such as coagulopathy, fluid shifts, prolonged inflammation, impaired host defense and metabolic acidosis (Martini et al., 2005; Asfar et al., 2014; Geurts et al., 2014). An attempt to bypass these side effects is pharmacological “metabolic inhibition” (Webb et al., 1966). This is of particular interest, since it has been hypothesized, that organ dysfunction in the critically ill organism may represent an adaptive metabolic downregulation in order to preserve organ function (Singer et al., 2004). In fact, sepsis-associated cardiac dysfunction is known to reflect a hibernation-like state, to ensure cell survival by entering a quiescent state, rather than a deleterious consequence (Levy et al., 2005). Thus, organ function could possibly be sustained, by a reversible state of “metabolic dormancy,” induced by a reduction of the cellular metabolism, in particular the mitochondrial oxidative phosphorylation, to maintain ATP-homeostasis (Singer et al., 2004). This concept is supported by a reduction of the cellular energy metabolism present during seasonal hibernation, which is accompanied by the downregulation of energy metabolism-associated proteins, e.g., mitochondrial electron transport chain complexes, acetyl CoA biosynthesis, Krebs cycle and glycolysis (Quinones et al., 2016).

The notion of a therapeutic “on-demand” suspended animation is exciting but fundamental questions remain: (1) How can a hibernation-like state be induced by reducing the energy demand of a complex organism? (2) How will the organism re-balance its energy demand in order to function in a suppressed metabolic state? The mitochondrial respiratory chain would be suitable, since mitochondria are the main cellular energy generators, and are targeted by gasotransmitters. This notion experienced a booming interest, when Blackstone et al. modulated energy metabolism of mice via H_2_S (Blackstone et al., 2005). The other gasotransmitters CO (Nystul and Roth, 2004) and NO (Teodoro and O'Farrell, 2003) were also applied with this purpose, and only recently, H_2_Se (Iwata et al., 2015) was reported to evoke similar effects.

This review discusses the role of gasotransmitters in inducing a suspended animation-like state, with particular focus on the modulation of mitochondrial function. Due to the complexity of this topic, we have mainly focused on the mitochondrial respiratory chain, as a functional entity of the mitochondria, and have only briefly addressed potential parallel intramitochondrial mechanisms.

## Nitric oxide (NO)

**Figure 2:**
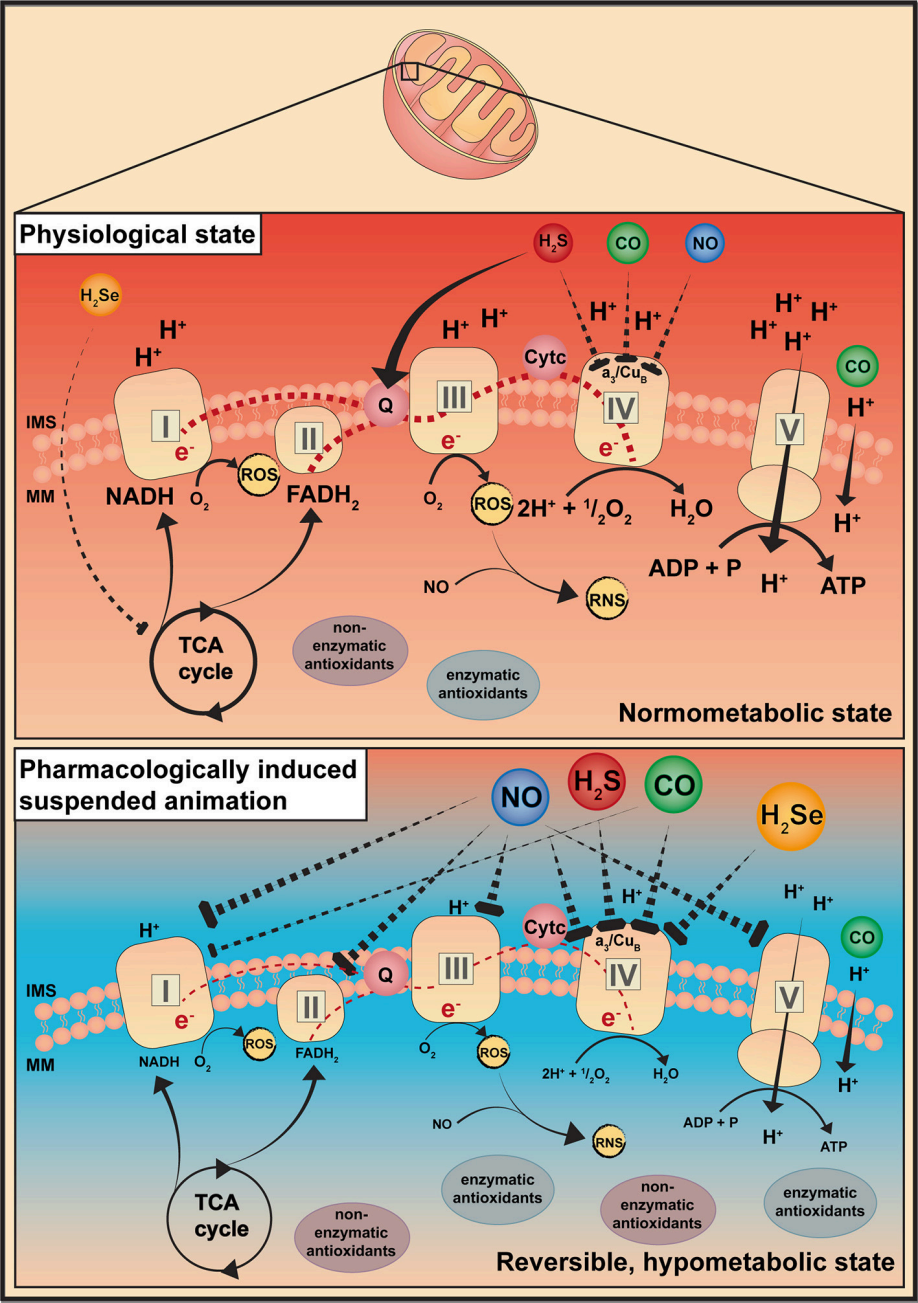
Gaseous mediators-induced suspended animation. This simplified illustration describes a possible explanation for a pharmacologically induced suspended animation by gaseous mediators. It is known that gaseous mediators are able to interact with the mitochondria, thereby modulating their bioenergetic function. Depending on the dose, gaseous mediators are supposed to specifically and reversibly inhibit the respiratory chain at distinct sites. CO, carbon monoxide; H_2_S, hydrogen sulfide; H_2_Se, hydrogen selenide; IMS, intermembrane space; MM, mitochondrial matrix; NO, nitric oxide; RNS, reactive nitrogen species; ROS, reactive oxygen species; TCA cycle, Krebs cycle.

NO and its derivatives (reactive nitrogen species; RNS) profoundly affect mitochondrial respiration under (patho-)physiological conditions (Figure 1). The impact of NO on mitochondrial respiration is dose-dependent, at low nanomolar concentrations NO inhibits the terminal enzyme of the mitochondrial respiratory chain, cytochrome c oxidase (COX), in a rapid, potent, selective, but also reversible manner (Figure 2). Unlike cyanide, mitochondrial respiration is not completely blocked as cells are still able to consume O_2_ and generate ATP, however at a reduced level. In contrast, the mitochondrial respiratory chain is slowly, non-selectively and irreversibly inhibited at high NO concentrations and upon NO-radical formation, e.g., peroxynitrite (ONOO^−^) (Brown and Borutaite, 2004). Sepsis-related changes in mitochondrial bioenergetics seem to be driven by rising NO concentrations, or by its metabolites (e.g., ONOO^−^) upon the reaction of NO with O_2_ radicals (Brealey et al., 2002; Singer et al., 2004). However, this NO-induced fall in metabolic rate may ultimately preserve mitochondrial respiration (Singer et al., 2004). In line with this, in porcine hemorrhagic shock low-dose intravenous sodium nitrite (11 mg) (and 250 ppm CO inhalation) preserved mitochondrial function, thus improving organ function (Haugaa et al., 2015). Due to its potential of reversibly inhibiting the mitochondrial respiratory chain, NO could theoretically induce an “on-demand” hibernation. To date, NO-induced suspended animation has been reported in *drosophila* during hypoxia (Teodoro and O'Farrell, 2003). NO mediated the shutdown of the cellular metabolism in response to hypoxia, thereby improving survival (Teodoro and O'Farrell, 2003). More recent studies reported, that NO was required for the beneficial effects of therapeutic hypothermia, thus NO inhalation improved outcome after cardiopulmonary resuscitation in hypothermia-treated mice (Kida et al., 2014).

## Carbon monoxide (CO)

Low CO concentrations exert anti-inflammatory and cytoprotective effects (Thiemermann, 2001) (Figure 1). In line with this, beneficial effects of inhaled CO (100–300 ppm), or CO-releasing donors (e.g., CORMs) have been reported during hemorrhagic shock in both small and large animals (Gomez et al., 2015), and/or acute lung injury (ALI) of various origin. The underlying mechanism comprises alterations of cell energy utilization, and preservation of mitochondrial function, e.g., increased anti-oxidative capacity and modulation of reactive oxygen species (ROS) signaling (Lee et al., 2011; Gomez et al., 2015). Briefly, CO-induced ROS signaling exerts cytoprotection and anti-inflammation via mitogen-activated protein kinase (MAPK) (Otterbein et al., 2000), regulates vasomotor response in vascular smooth muscle cells but also O_2_ sensing by the interaction of CO with Ca^2+^-dependent K^+^-channels (Riesco-Fagundo et al., 2001) and exerts anti-proliferative effects through modulating NADPH oxidase activity (Taille et al., 2005) (Figure 2). Protective effects were, however, not reproducible in human endotoxemia, despite similar carboxyhemoglobin (COHb) levels (Mayr et al., 2005). In contrast, high CO concentrations are cytotoxic owed to the irreversible inhibition of COX activity by targeting Fe^2+^ of reduced heme moieties, thereby outcompeting O_2_ (Stamler and Piantadosi, 1996; Rose et al., 2017), but also due to increased radical stress production, secondary to the respiratory chain inhibition and subsequent tissue damage (Ischiropoulos et al., 1996) (Figure 2). However, unlike other gasotransmitters, the binding of CO to COX is highly dependent on O_2_ levels and, hence, can be dissociated by hyperoxia, in particular at supra-atmospheric pressures (Rose et al., 2017).

Low CO concentrations are known to reversibly decrease COX activity, thereby reducing mitochondrial O_2_ consumption. This would represent a reasonable leverage point to artificially inhibit mitochondrial respiration. However, reducing COX activity inevitably accumulates electrons at complex III, thereby generating low amounts of ROS. It is known that ROS production is a common byproduct of mitochondrial oxidative metabolism, representing 1–2% of O_2_ consumption, and, that ROS can function as a signaling factor. As the biological functions of CO, such as the modulation of mitochondrial bioenergetics and cytoprotection, are dependent on mitochondrial ROS signaling, CO precisely controls cellular ROS levels by inducing electron leakage over the mitochondrial inner membrane, thereby promoting a mechanism referred to as mild uncoupling (Lo Iacono et al., 2011). However, other studies even showed, that CO improved COX activity, possibly due to a two-step response, with a transient initial decrease and later increase of mitochondrial respiration including COX activity (Queiroga et al., 2011). Besides, the competing binding of CO and NO to heme irons in proteins may promote the liberation of NO. When interacting with the mitochondrial respiratory chain, CO also promotes the liberation of ${\text{O}}_{2}^{-}$. Their simultaneous liberation can ultimately generate OONO^−^, which in turn may aggravate nitrosative and oxidative stress (Stamler and Piantadosi, 1996). Nevertheless, CO-induced suspended animation was performed in *caenorhabditis elegans* (Nystul and Roth, 2004). Although theoretically, the binding properties of CO and its possibility of dissociation *via* hyperoxia make CO a promising candidate, some issues need further investigation. (1) CO tolerance is species-dependent (e.g., for human compared to rodents), and hence, the most efficient and least harmful doses for human CO administration remain to be determined. Nevertheless, clinical trials are currently investigating different routes of administration and doses in order to assess possible beneficial effects, e.g., during acute respiratory distress syndrome (ARDS) (NCT02425579), and to bypass CO-related toxicity due to accumulating CO levels following continuous application. (2) Excessive ROS production during critical illness, could mitigate ROS-dependent biological functions of CO, promote tissue damage, aggravate mitochondrial uncoupling, eventually leading to mitochondrial dysfunction. (3) Due to competitive binding of O_2_ and CO to COX, CO-mediated mitochondrial inhibition is O_2_ dependent, and thus, more pronounced under hypoxic conditions. Therefore, any clinical use of CO must be cautioned under conditions of tissue hypoxia.

## Hydrogen sulfide (H_2_S)

It is well established that H_2_S dose-dependently affects mitochondrial bioenergetics. In contrast to NO and CO, H_2_S also feeds electrons into the electron transport chain (ETC) of mammalian cells at the level of coenzyme Q via sulfide quinone reductase (SQR) (Lagoutte et al., 2010) (Figure 2). In slightly higher concentrations, however, H_2_S reversibly binds to COX, and this seems to be the underlying mechanism involved in the landmark study by Blackstone et al. (2005) reporting that awake and spontaneously breathing mice, inhaling subtoxic H_2_S concentrations (20–80 ppm), reversibly decreased their energy expenditure, which coincided with a fall in core temperature. Moreover, Volpato et al. reported that the reduced metabolic activity went along with bradycardia and a reduced cardiac output but well maintained stroke volume and blood pressure (Volpato et al., 2008). Further, H_2_S exerted organ-protective effects by inducing a hypometabolic state and preserving mitochondrial function (Elrod et al., 2007; Bos et al., 2009), ultimately improving survival (Morrison et al., 2008), in various shock models. In mice, inhaled H_2_S (100 ppm) in combination with hypothermia allowed better maintenance of liver mitochondrial integrity and switched energy metabolism to increased glucose oxidation (despite identical total energy expenditure) (Baumgart et al., 2010). Usually, energy metabolism of living animals is supported by carbohydrate (glycolysis) and lipid oxidation (β-oxidation). A switch in fuel utilization toward increased glucose oxidation improves the yield of oxidative phosphorylation due to a higher ATP synthesis/O_2_ consumption ratio for glycolysis than for β-oxidation, as the electon donor nicotamide adenine dinucleotide (NADH) provides three coupling sites rather than two coupling sites from flavin adenine dinucleotide (FADH_2_) (Leverve, 2007).

However, H_2_S could pose problems during clinical application, because (1) Gaseous H_2_S induces irritation to the respiratory tract (Simon et al., 2008). Thus, in order to bypass these adverse effects, current studies are investigating soluble, slow-releasing H_2_S-donors, in particular commercially available compounds, which are already clinically approved, e.g., thiosulfate or ammonium tetrathiomolybdate (Dyson et al., 2017). (2) The metabolic depression could not be reproduced in anesthetized and mechanically ventilated mice under normothermic conditions (Baumgart et al., 2010). (3) Any H_2_S-related reduction in metabolic activity seems to depend on the species size regardless of the route of administration (Simon et al., 2008; Asfar et al., 2014). In line with this, inhaling 10 ppm H_2_S during exercise decreased O_2_ uptake in healthy volunteers, most likely due to toxic reduction of maximal aerobic capacity rather than to a regulatory effect on mitochondrial respiration (Bhambhani et al., 1997). (4) H_2_S-induced inhibition is O_2_ and temperature dependent, thus increasing the risk of toxic effects upon hyperthermia and/or tissue hypoxia (Gröger et al., 2012; Matallo et al., 2014).

## Hydrogen selenide (H_2_Se)

Iwata et al. reported the induction of a suspended animation-like state with H_2_Se (Iwata et al., 2015). Mice, inhaling low concentrations of H_2_Se (5 ppm), reduced their energy expenditure as documented by reduced carbon dioxide (CO_2_) production and O_2_ uptake (Iwata et al., 2015). These effects resemble the effects of inhaled H_2_S (Blackstone et al., 2005), and the authors suggested that H_2_Se may also induce reversible COX inhibition, in fact, the oxidation product of selenide, selenite, binds to COX (Figure 2). Moreover, low selenite concentrations preserved organ function by slowing down glutamate and malate-stimulated mitochondrial respiration (i.e., complex I), eventually resulting in improved mitochondrial capacity and biogenesis in I/R injury (Mehta et al., 2012). Iwata et al. also demonstrated that the injectable selenide preparation NaSeH attenuated post-ischemic tissue injury in murine myocardial ischemia, by preferentially targeting the injured tissue (Iwata et al., 2015). However, apart from the lack of translation to larger species, H_2_Se entails considerable risks, due to a narrow therapeutic range and a pronounced environmental hazard.

## Conclusion

Pharmacological induction of a hypometabolic state, to hibernate the whole organism and/or just isolated organs, is a highly attractive option for critical care or transplant medicine. The use of gaseous mediators has attracted great interest, and multiple studies have been published supporting the concept of “Buying time in suspended animation.” Nevertheless, despite impressive and promising data, multiple questions have aroused in respect to its feasibility in humans. Albeit being endogenously produced, they may irreversibly inhibit the mitochondrial respiratory chain, which would outweigh any potential benefits. Therefore, slow-releasing donors, in particular compounds already recognized for other indications, may help to bypass severe and long-term damage.

## Author contributions

Substantial contribution to the conception of the work, drafting work, final approval, and agreement to be accountable for all aspects of the work: CH, PR, BN, EC, and MW. Design of figures: CH and MW.

### Conflict of interest statement

The authors declare that the research was conducted in the absence of any commercial or financial relationships that could be construed as a potential conflict of interest.
